# Design and Development of a Hospital-Based Coronary Artery Disease (CAD) Registry in Iran

**DOI:** 10.1155/2023/3075489

**Published:** 2023-01-25

**Authors:** Ali Garavand, Reza Rabiei, Hassan Emami

**Affiliations:** ^1^Department of Health Information Technology, School of Allied Medical Sciences, Lorestan University of Medical Sciences, Khorramabad, Iran; ^2^Department of Health Information Technology and Management, School of Allied Medical Sciences, Shahid Beheshti University of Medical Sciences, Tehran, Iran

## Abstract

**Background:**

The incidence of coronary artery disease (CAD), the leading cause of mortality in most developed and developing countries, is increasing. The adoption of hospital registries can improve care delivery and facilitate the management of CAD through better planning, as well as help with outcome assessment through more effective data management.

**Objectives:**

The present study is aimed at designing a hospital-based CAD registry for managing CAD data.

**Methods:**

This developmental study was conducted in three phases. Initially, sources related to CAD registries were reviewed, the results of which were published in two studies. In the next phase, the prerequisites and requisites of the software were determined through a qualitative study. In this phase, the registry dataset was determined by using a questionnaire. Finally, the developed conceptual model of the software was validated. The software was then developed based on the validated conceptual model.

**Results:**

The registry data elements were classified into 13 main categories, including identification data, medical history, and risk factors. The dataset included 171 data elements, including data related to surgical and nonsurgical procedures. The conceptual model was approved by field experts, and the software was developed accordingly.

**Conclusion:**

The steps followed in the present study for developing the CAD registry can be used as an appropriate approach for designing similar hospital-based registries. Considering the pivotal role of the registry in the management of CAD, the routine and systemic use of the registry is suggested in all healthcare centers.

## 1. Introduction

Cardiovascular diseases are a group of disorders affecting the heart or blood vessels. They are classified into different types, including coronary artery disease (CAD), heart attacks, and rheumatic heart disease, according to the affected site, conditions, and contributing factors [1, 2]. One of the most important types of cardiovascular disease is CAD [3] scientifically known as coronary heart disease, ischemic heart disease, or atherosclerosis [4]. CAD or ischemic heart disease is caused by the narrowing or spasm of coronary arteries, which are the main vessels supplying blood to the heart, resulting in the reduction of blood and oxygen supply to the heart muscles; consequently, it exposes the individual to heart attacks and other complications [2, 5].

As a chronic disease, CAD has been introduced as the leading cause of mortality worldwide, especially in developed countries; today, it still accounts for the highest number of deaths globally [2, 6, 7].

The report of the World Health Organization in 2020 shows that cardiovascular diseases, as one of the noncommunicable diseases, are the most important cause of death across the world [8]. In 2015, about 17.7 million deaths were reported worldwide due to cardiovascular diseases, and the highest death rate was assigned to coronary artery disease [9]. According to the Center for Disease Control and Prevention (CDC) in the United States, 610,000 people die each year due to cardiovascular disease [10]. In Iran, as a developing country with a low-income level, 26.4% of recorded deaths were due to cardiovascular diseases [8, 11].

The need for increased attention and planning to deal with the complications of CAD is strongly felt, especially in developing countries. Although the World Health Organization (WHO) reported that more than 80% of countries in the world have the necessary resources and facilities to deal with noncommunicable diseases, including cardiovascular diseases [12, 13], they (especially cardiovascular diseases) remain the leading cause of death in different communities. Therefore, it is necessary to apply all the available facilities and potentials to manage this disease, including CAD data management systems, to effectively manage complications and consequences through proper planning [14, 15].

The management of disease-related data can be regarded as a basis for achieving various healthcare goals and implementing relevant programs [16, 17]. Registries are one of the most important patient data management tools that play a critical role in managing diseases and conducting relevant research [17]. Disease registries present a continuous and systematic collection of information about individuals with specific diseases or health conditions in a given population [16–18]. By applying clinical guidelines and data standards, these registries can reduce the costs of care and improve care delivery [19–22].

Owing to the great impact of CAD on communities and the special attention of authorities in developed countries to the management and control of this disease, different types of CAD registries have been developed in different countries [23, 24]. For example, the Swedish Coronary Angiography and Angioplasty Registry (SCAAR) was developed in 1999 in Sweden by merging the Swedish Coronary Angiography Registry (Acta Coronaria) with the Swedish Registry for Coronary Angioplasty (SCAP) as part of the Swedish Heart Registry. This registry has been in place in a web-based format since 2001, and the data are collected online from 30 hospitals [25–27].

Considering the advantages of registry systems as data management tools, such as prevention of financial loss, data documentation, improvement of healthcare planning, delivery of high-quality information to researchers, improvement of healthcare quality, and assessing the outcomes of care [28–30], there is a need for CAD registry design and deployment. Therefore, the present study is aimed at designing a hospital-based CAD registry software in Iran.

## 2. Methods

This applied that the developmental study was conducted in three phases.

### 2.1. Phase 1

This step was conducted in two parts: conducting a systematic review of studies focusing on the key processes of registry software systems and performing a comparative study of the selected registries using a data management approach, the results of which have been published earlier [31, 32].

### 2.2. Phase 2

Initially, for software requirement elicitation, a qualitative study was conducted by interviewing 15 potential users, including the software administrator, data processing expert, cardiologist, and cardiac nurse. In the next stage, the minimum dataset for the hospital-based CAD registry was created by reviewing the patient's records, and the software processing indicators were presented in the form of a questionnaire, and the opinions of cardiologists (*n* = 6), cardiac nurses (*n* = 9), and health information management specialists (HIM) (*n* = 6) were sought.

For this aim, this part of the study was designed and implemented as a Delphi study. Data collection was performed using a questionnaire containing the minimum dataset for the CAD hospital registry. In the first round of Delphi, the questionnaire developed on a five-point Likert scale was given to the participants, and they were asked to state their level of agreement with each data item in the software. In this round, at the end of each part of the questionnaire, the participants were provided with an open question that allowed them to express their views if the closed questions did not address these appropriately, and these were added to the questionnaire for the next round. In this stage, there were 166 data items for the minimum dataset, and 20 indicators were set for the reporting function of the software. In this round of the Delphi study, two data items were removed, seven data items were added, and five items were passed to the second round of study. In the first round, all the indicators in the questionnaire were approved by participants. In the second round, the questionnaire contained 12 data items, and it was passed to participants involved in the first stage. Eleven out of 12 items were approved by participants.

The inclusion criteria in this stage were willingness to participate in the study and having at least five years of relevant work experience. Data analysis was performed in SPSS 22 by measuring descriptive statistics, including mode, median, mean, interquartile range, and percentage of agreement.

### 2.3. Phase 3

In this stage, the operational, structural, and behavioral models of the software were designed using Enterprise Architect version 9, based on the features and requirements of CAD hospital registries. Seven experts in the fields of health information management, medical informatics, and software engineering, with at least five years of work experience, participated in this study to determine the validity of the presented conceptual model of the software. Data analysis was performed in SPSS 22 by measuring descriptive statistics. Diagrams with scores above 75% (12 out of 16) were approved, while diagrams with scores below 75% were reviewed. After approving the conceptual model, the CAD hospital registry software was designed.

MySQL was also used for developing the software database, and Visual Asp.Net 2013 programming language was applied for software development using C#, JQuery, CSS, Ajax, and HTML5 languages. Error detection and debugging were carried out after coding and developing the initial version in which the syntax errors were checked and fixed. The semantic errors of the codes were then checked and eliminated. After fixing the syntactical and semantic errors, the logical errors of the software were also checked to make sure about the functionality of the developed software.

## 3. Results

### 3.1. Phase One

The results of the first phase of this study were published in our two previous articles, as indicated earlier [31, 32]. Dealt with the registry data items (13 main categories), registry users, key registry indicators, key organizations and individuals using registries, status, goals of patient follow-up, and control of registry data quality were reported.

### 3.2. Phase Two

In the second phase conducted to identify the software requirements, 15 cardiologists, cardiac nurses, and data processing experts participated in a qualitative study.  Table 1 shows the demographic information of participants in the stage of the study. At this stage, 60% of the study sample was female (*n* = 9), and the participation of health information management specialists in the interviews was more than the other groups (33.33%).

Table 2 presents the operational requirements of the software, based on the key registry processes divided into six main categories.

#### 3.2.1. Determination of the Minimum Dataset and Indicators of the Hospital-Based CAD Registry

To determine the minimum dataset, besides conducting a systematic study and examining the patients' files, the data resulting from the Delphi technique were presented to experts. The first Delphi round was conducted to determine the minimum dataset for the CAD registry software. Accordingly, the minimum dataset of the CAD registry was divided into 13 main categories (Table 3). In the supplementary file (available here) of the article, all data items have been presented.

In the first phase of the Delphi, factors, including the number of children, patient referral to the hospital (e.g., admission from another hospital, outpatient admission, emergency admission by ambulance, and admission based on code 247), obesity status based on the body mass index (BMI), type of narcotic drug (morphine and pethidine), the onset of symptoms before the first emergency call, onset of symptoms until referral to the center, and angiography indication class, were suggested by participants to be included into the questionnaire for the second round.

In the second round of the Delphi, except for the results of follow-ups and outcomes after discharge, other items were approved. Overall, 171 information fields were finalized in two Delphi rounds for the minimum dataset of the hospital-based CAD registry software. Moreover, the software reporting indicators were agreed upon by experts in the first round of Delphi, as presented in Table 4.

### 3.3. Phase Three

The conceptual model included operational, structural, and behavioral parts, as discussed in the following section.

Based on our investigations, the system users included the software administrator, cardiologist, cardiac nurse, and data processing experts. To design the operational model, relevant scenarios were first prepared, such as the creation of a user account, where the software administrator defines a new user account and determines the user's role, allowing the user to login by entering their login details. Other scenarios were also prepared based on the main services of the software, including new case registration, abstracting, and reporting scenarios.

After developing the operational model, the structural model of the software was designed covering the general class diagram of the software.

In general, various diagrams are used to demonstrate a software behavioral model. The sequence diagram presents a comprehensive description of the main applications of the software and facilitates software development. A sequence diagram was created and validated based on the main applications of the software, including case finding, data collection, abstracting, data quality control, follow-up, and reporting.

Table 5 illustrates the validity results of the CAD registry conceptual model.

**Figure 1:**
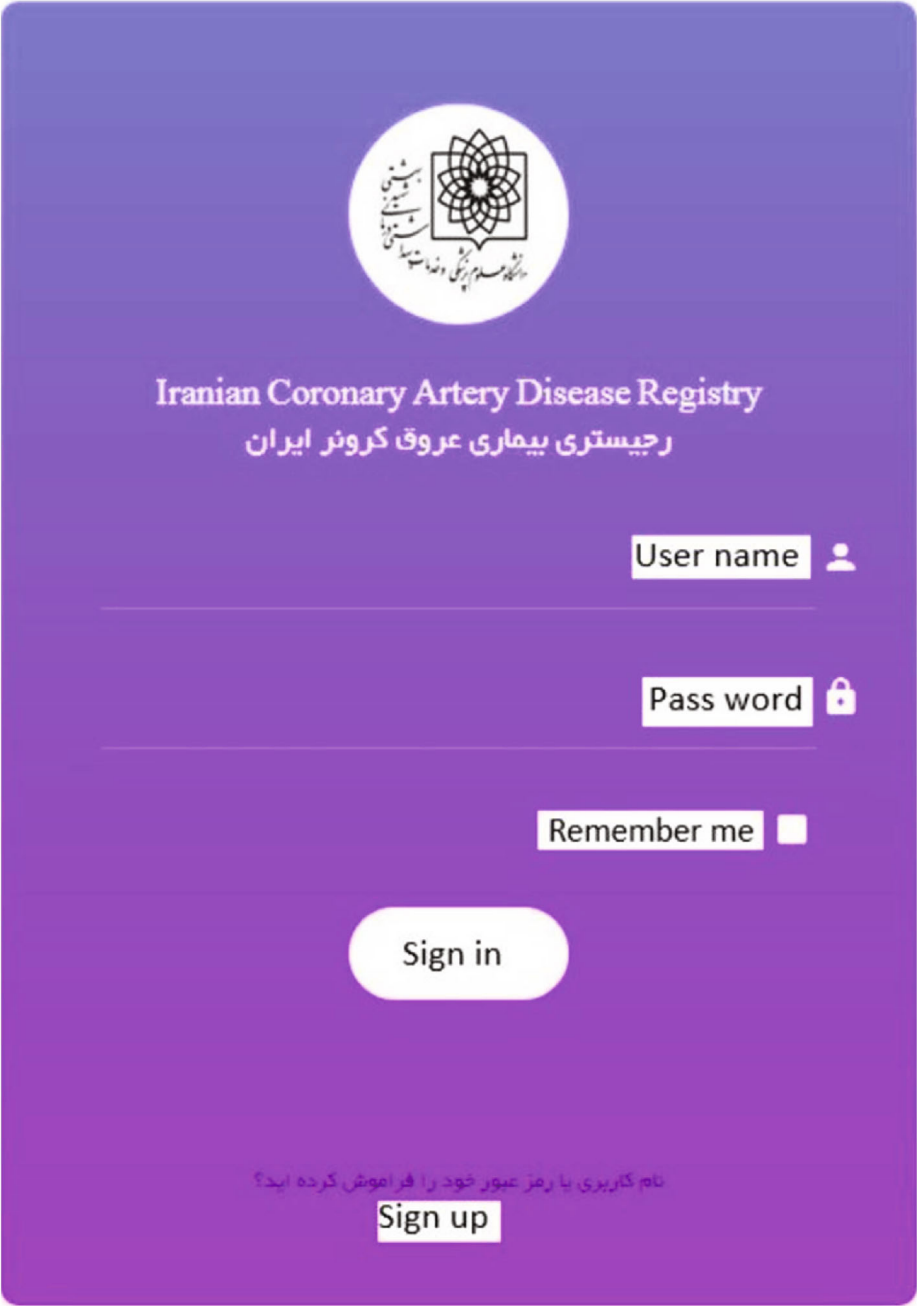
The first page of the software.

According to the table above, a score above 75% approved each diagram; all relevant diagrams were confirmed by experts.  Figure 1 demonstrates the first page of the software.

**Figure 2:**
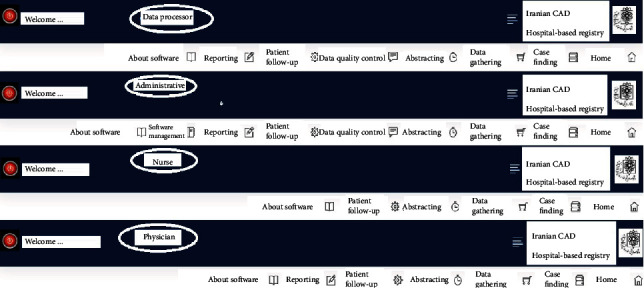
User interface and user access level.

Figure 2 shows the user interface and user access level of the software.

The users could be able to select any type of report from reporting function of the registry and obtain their needed reports.

## 4. Discussion

A key step in developing information systems is the identification of operational and nonoperational requirements (software prerequisites) through users' need assessment [33, 34] [27, 28]. The practical principles of software design and assessment (i.e., system development life cycle, including software planning, analysis, design, and implementation) were applied in the present study. The operational requirements of the hospital-based CAD registry software were determined based on the key registry processes, which included case finding, data collection, abstracting, data quality control, patient follow-up, and registry software reporting.

The findings showed that the hospital-based CAD registry software should be capable of registering newly identified cases of CAD based on the existing information facilities and infrastructure. Effective registries are required to include all new cases of the target disease [31, 35]. In well-developed registries, new case finding techniques, such as text mining, are applied; however, if these techniques are not addressed automatically, data processing experts should actively check for relevant sources and find new cases in the registry. Considering the current status of health information systems in Iran, the evaluation of hospital information systems and case finding step is necessary, as the goal of case finding in registries (from the experts' perspectives in this study) is to avoid errors and help the users find all relevant cases.

From the perspective of experts in this study, the CAD registry software should be capable of requesting case finding information and recording a summary of data from the patient's records. Moreover, the software should be capable of storing data temporarily in a temporary file if the information of a new case is unavailable. In a previous study, Tabrizi et al. also emphasized the importance of creating a temporary file [36]. Generally, the most important feature of a CAD registry system is data abstraction, documentation of all necessary information, and prevention of recording irrelevant or unimportant data [36–38].

Moreover, the results of the present study showed that data quality control should be a key operational function in the software. Data quality control ensures that the system is capable of checking data quality attributes [39] and could support users in this matter. Therefore, a hospital-based CAD registry should support data quality control and indicate high-quality from low-quality abstracts. Another operational capacity of our CAD registry software was patient follow-up. From the perspective of experts, the software should be capable of providing a list of patients who require follow-up regularly. Although there are more advanced methods, such as sending SMS and email to patients for follow-ups [40, 41], phone calls can help to make sure that patients are updated appropriately about their follow-ups.

Other results of the present study showed that the registry software should be capable of preparing different reports that should be sent to relevant bodies. According to the findings, the software should be allowed to calculate important indicators of the disease and provide reports in formats, such as PDF and Excel files needed by users. Overall, determining the minimum dataset for health information systems, especially disease registries, could help to make sure that required data are collected and prevents unnecessary data collection [42, 43]. This stage is a key step in developing disease registries [44, 45]; therefore, in this study, the minimum dataset of the CAD registry was developed, in which data items fell into 13 main categories, including demographic information, insurance information, hospitalization information, medical history, risk factors, medications and laboratory test results, physician examination results, angiography findings, data related to noninvasive procedures or invasive and surgical procedures, patient's status during discharge, and patient follow-up.

In the current study we use of object-oriented approach for designing the conceptual model of the software. Scenarios were initially created, and the conceptual model was developed and composed of operational, structural, and behavioral component parts. The object-oriented approach provides a comprehensive view of the design and development of information systems and tries to model the whole system considering different parts of the system in line with expected functionality. This approach could help developers to obtain a better understanding of users' needs [36]; in other words, the object-oriented approach is a software programming process that takes advantage of object interactions to solve a problem [46].

In the present study, a standard integrated language was used to develop the conceptual model. Designs based on the object-orientated concepts in the Unified Modeling Language (UML) make them fully compatible with object-oriented programming environments and languages (e.g., C++, Java, and C#), and UML has become the common language of many developers of information systems [46, 47]. Besides, UML has been widely used in the development of disease registries [47]. In a previous study, Nouri developed a registry based on a study focusing on hemoglobinopathy using UML [48]. Therefore, it is suggested to use this standard and international language in developing conceptual disease registry models.

A key limitation of the current study could be developed in the CAD registry at a hospital-based level, while a population-based registry could provide a more robust basis for controlling the CAD in a given population. However, the hospital-based CAD registry developed in the current study could be a basis for developing a population-based registry. In addition, the duration of the pilot registry was 6 months, and a longer period is required to depict a more realistic picture of the developed registry and figure out its capabilities and limitations over time.

## 5. Conclusion

Based on the results of the present study, paying attention to data collection and abstracting is critical in developing disease registry software. With respect to patient follow-ups, although patients could be informed about their attendance using SMS and emails, phone calls for the follow-up of patients with CAD seem to be a more appropriate option, because, in the event of a patient's death, the data processing expert can obtain further information, such as the place of death, cause of death, and date of death through a phone call.

## Figures and Tables

**Table 1:** Characteristics of participants in the qualitative phase of the study.

Demographic information		Frequency	%
Sex	Women	9	60
	Men	6	40

Type of specialty	Physician	4	26.67
	Nurse	4	26.67
	HIT expert	2	13.33
	HIM specialists	5	33.33

Academic level	MSc	4	26.67
	Ph.D.	7	46.66
	MD	4	26.67

**Table 2:** The operational requirements of the CAD registry software.

Operational requirements	Description	Sources
Case finding	The registry software should allow the registration of newly identified cases.	Interviews 7, 8, and 13
Data collection	The software should be capable of requesting information about the case they found.	Interviews 3, 10, 12, and 14
Abstracting	The software should allow the recording of a summary and abstract of data.	Interviews 2, 7, and 9
Data quality control	The software should be capable of performing data quality control and identifying high-quality from low-quality abstracts.	Interviews 4, 5, and 11
Follow-up	The software should be capable of providing a list of patients who require regular follow-up.	Interviews 9, 10, and 15
Reporting capacity	The software should be capable of creating different reports, and if needed, sharing these reports with qualified care centers and individuals.	Interviews 11 and 14

**Table 3:** The results of the first Delphi round for the minimum dataset of the CAD registry software.

Data elements		Mean	No. of items	No. of accepted items
Demographic information		4.11	15	14
Insurance information		3.95	4	4
Hospitalization information		4.00	5	4
Medical history and risk factors	History of other diseases	4.25	18	18
	Lifestyle	4.55	5	5
Medications		4.51	14	14
Laboratory test results		4.80	23	23
Physician examination results	Present symptoms	4.30	10	10
	CAD type detection	4.90	1	1
Information on angiography		4.57	7	7
Noninvasive procedures	CT angiography	4.61	2	2
	Other	4.29	3	3
Information on invasive and surgical procedures	General	4.26	5	5
	Determination of the type of PCI procedure and related information	4.52	8	8
	Outcomes	4.27	13	13
Patient's status during discharge		4.01	5	4
Patient follow-up	Follow-up results and outcomes after discharge, up to six months	4.43	9	8
	Follow-up of outcomes due to coronary artery disease	4.41	6	6
	Follow-up of patient's adherence to treatment	3.79	5	4
	Follow-up of patient's quality of life	3.98	3	3
	Follow-up of death status	3.69	5	3

**Table 4:** Reporting indicators required in the software.

Rows	Indicators	Mode	Median	Mean	Interquartile range		Percentage of agreement	Results
					Q1	Q3		
1	Number of patients with coronary artery disease (CAD)	3	4	3.81	3	5	76	✓
2	Number of patients by sex	4	4	4.33	4	5	86.6	✓
3	Physician's performance	5	5	4.57	4	5	91.4	✓
4	Mean age of patients (total age of all patients/total patients)	5	5	4.76	4.5	5	95.2	✓
5	Mean length of hospital stay of patients (number of hospitalization days of all patients/total patients)	5	5	4.57	4	5	91.4	✓
6	The ratio of patients diagnosed with STEMI to patients diagnosed with non-STEMI (number of patients with STEMI/number of non-STEMI patients)	5	5	4.42	4	5	88.4	✓
7	Death rate	5	5	4.57	4	5	91.4	✓
8	Patients with a history of cardiovascular surgeries	5	5	4.71	4	5	94.2	✓
9	Comparison of the status of patients during discharge, including the number of recovered patients, number of nonrecovered patients, the ratio of recovered to nonrecovered patients, and the number of patients discharged at their discretion	5	5	4.57	4	5	91.4	✓
10	Death rate (number of patients who expired/total number of patients in the specified setting)	5	5	4.43	4	5	88.6	✓
11	Total number of PCIs performed	4	4	4.33	4	5	86.6	✓
12	Number of follow-ups	5	5	4.57	4	5	91.4	✓
13	Comparison of echocardiography indicators	5	5	4.57	4	5	91.4	✓
14	Mean time from the onset of symptoms until referral to the hospital	5	5	4.52	4	5	90.4	✓
15	Frequency and ratio of patient referral to the hospital	5	5	4.38	3.5	5	87.6	✓
16	Comparison of involved vessels (e.g., calculation of the frequency and ratio of involved vessels)	4	4	4.09	3	5	81.8	✓
17	Type of angiography performed	4	4	3.82	2.5	5	76.4	✓
18	Calculation of the degree of vessel occlusion	5	5	4	3	5	80	✓
19	Calculation and comparison of the indication class	5	4	3.95	3	5	78	✓
20	The rate of use of each angiography method	3	4	3.81	3	5	76	✓

**Table 5:** Validation results of the CAD registry conceptual model.

Diagram titles		Acceptable		Somewhat acceptable		Unacceptable		Score	Percentage	Diagram acceptability results
		Frequency	Percentage			Frequency	Percentage			
				Frequency	Percentage					
Diagram of the software	Log in	8	100	0	0	0	0	16	100	✓
	User account creation	8	100	0	0	0	0	16	100	✓
	Case finding	8	100	0	0	0	0	16	100	✓
	Data collection	7	87.5	1	12.5	0	100	15	93.75	✓
	Abstracting	7	87.5	1	12.5	0	100	15	93.75	✓
	Data quality control	8	100	0	0	0	0	16	100	✓
	Reporting	7	87.5	1	12.5	0	100	15	93.75	✓
	Patient follow-up	8	100	0	0	0	0	16	100	✓
	Log out	8	100	0	0	0	0	16	100	✓

Software activity diagram	Log in	8	100	0	0	0	0	16	100	✓
	User account creation	8	100	0	0	0	0	16	100	✓
	Case finding	8	100	0	0	0	0	16	100	✓
	Data collection	7	100	0	0	0	0	16	100	✓
	Abstracting	6	75	1	12.5	1	12.5	13	81.25	✓
	Data quality control	8	100	0	0	0	0	16	100	✓
	Reporting	7	87.5	1	0	1	12.5	14	87.5	✓
	Patient follow-up	8	100	0	0	0	0	16	100	✓
	Log out	8	100	0	0	0	0	16	100	✓

Software class diagram		6	75	2	25	0	0	14	87.5	✓

Software sequence diagram	Case finding	8	100	0	0	0	0	16	100	✓
	Data collection	7	87.5	0	0	1	12.5	14	87.5	✓
	Abstracting	6	87.5	1	12.5	1	12.5	13	81.25	✓
	Data quality control	8	100	0	0	0	0	16	100	✓
	Reporting	7	87.5	1	12.5	0	0	14	87.5	✓
	Patient follow-up	8	100	0	0	0	0	16	100	✓

## Data Availability

All data of the study were reported. The link of the registry is https://cad.hisapps.ir/User/Login?ReturnUrl=%2f.
