# Supplementary material for: Design and Development of a Hospital-Based Coronary Artery Disease (CAD) Registry in Iran
Source: Biomed Res Int. 2023 Jan 25;2023:3075489. doi: 10.1155/2023/3075489 (PMC9891832; doi:10.1155/2023/3075489)
Supplement: Supplementary Materials — Supplemental material for this article is available online. Supplementary material file 1: all data items of the CAD registry in the first round of Delphi. [file 3075489.f1.docx]

Supplementary file 1: All data items of the CAD registry in the first round of Delphi

| **Rows** | **Data elements** | | | | | | **Mode** | **Median** | **Mean** | **Interquartile range** | | **Percentage of agreement** | **Result** |
| --- | --- | --- | --- | --- | --- | --- | --- | --- | --- | --- | --- | --- | --- |
|  |  |  |  |  |  |  |  |  |  | **Q1** | **Q3** |  |  |
| 1 | Demographic information | | | | First name and last name | | 5 | 5 | 4.90 | 5 | 5 | 98 | 🗸 |
| 2 |  |  |  |  | Identification number | | 5 | 5 | 4 | 3 | 5 | 80 | 🗸 |
| 3 |  |  |  |  | National code | | 5 | 4 | 3.95 | 3 | 5 | 78 | 🗸 |
| 4 |  |  |  |  | Father’s name | | 4 | 4 | 3.86 | 2.5 | 5 | 76.4 | 🗸 |
| 5 |  |  |  |  | Sex | | 5 | 5 | 4.86 | 5 | 5 | 97.2 | 🗸 |
| 6 |  |  |  |  | Age | | 5 | 5 | 4.95 | 5 | 5 | 99 | 🗸 |
| 7 |  |  |  |  | Marital status | | 5 | 5 | 4.38 | 3.5 | 5 | 87.6 | 🗸 |
| 8 |  |  |  |  | Level of education | | 5 | 5 | 4.71 | 4 | 5 | 94.2 | 🗸 |
| 9 |  |  |  |  | Occupation | | 5 | 5 | 4.38 | 3.5 | 5 | 87.6 | 🗸 |
| 10 |  |  |  |  | Address | | 4 | 4 | 4.09 | 3 | 5 | 81.8 | 🗸 |
| 11 |  |  |  |  | Phone number | | 4 | 4 | 3.82 | 2.5 | 5 | 76.4 | 🗸 |
| 12 |  |  |  |  | Email | | 4 | 4 | 3.82 | 2.5 | 5 | 76.4 | 🗸 |
| 13 |  |  |  |  | Ethnicity | | 5 | 4 | 4.33 | 3.5 | 5 | 86.6 | 🗸 |
| 14 |  |  |  |  | Nationality | | 4 | 4 | 3.95 | 3 | 5 | 79 | 🗸 |
| 15 |  |  |  |  | Religion | | 1 | 2 | 1.76 | 1 | 3 | 35.2 | × |
| 16 | Insurance information | | | | Insurance status | | 4 | 4 | 3.82 | 2.5 | 5 | 76.4 | 🗸 |
| 17 |  |  |  |  | Insurance company | | 4 | 4 | 3.82 | 2.5 | 5 | 76.4 | 🗸 |
| 18 |  |  |  |  | Insurance code | | 4 | 4 | 4.09 | 3 | 5 | 81.8 | 🗸 |
| 19 |  |  |  |  | Features of supplemental insurance | | 4 | 4 | 4.09 | 3 | 5 | 81.8 | 🗸 |
| 20 | Hospitalization information | | | | Name and features of the hospital providing services | | 3 | 3 | 3.76 | 3 | 5 | 75.2 | 🗸 |
| 21 |  |  |  |  | Physician’s name | | 5 | 5 | 4.61 | 4 | 5 | 92.2 | 🗸 |
| 22 |  |  |  |  | Date of admission and discharge | | 5 | 5 | 4.09 | 3 | 5 | 81.8 | 🗸 |
| 23 |  |  |  |  | Reasons for referral and hospitalization | | 4 | 4 | 3.95 | 3 | 5 | 79 | 🗸 |
| 24 |  |  |  |  | Number of medical files (records) | | 3 | 3 | 3.60 | 3 | 5 | 72 | × |
| 25 | Medical history and risk factors | | History of other diseases | | History of high blood pressure | | 5 | 5 | 4.86 | 5 | 5 | 97.2 | 🗸 |
| 26 |  |  |  |  | Diabetes | | 5 | 5 | 4.95 | 5 | 5 | 99 | 🗸 |
| 27 |  |  |  |  | Dyslipidemia | | 5 | 5 | 4.38 | 3.5 | 5 | 87.6 | 🗸 |
| 28 |  |  |  |  | Stroke or transient ischemic attack | | 5 | 5 | 4.71 | 4 | 5 | 94.2 | 🗸 |
| 29 |  |  |  |  | History of peripheral vascular disease | | 5 | 5 | 4.38 | 3.5 | 5 | 87.6 | 🗸 |
| 30 |  |  |  |  | History of CCU hospitalization | | 4 | 4 | 4.09 | 3 | 5 | 81.8 | 🗸 |
| 31 |  |  |  |  | History of an interventional cardiac treatment | | 4 | 4 | 3.82 | 2.5 | 5 | 76.4 | 🗸 |
| 32 |  |  |  |  | History of myocardial infarction | | 4 | 4 | 3.82 | 2.5 | 5 | 76.4 | 🗸 |
| 33 |  |  |  |  | History of angina | | 5 | 4 | 4.33 | 3.5 | 5 | 86.6 | 🗸 |
| 34 |  |  |  |  | Cancer | | 3 | 3 | 3.76 | 3 | 5 | 75.2 | 🗸 |
| 35 |  |  |  |  | Type of cancer if diagnosed | | 5 | 5 | 4.61 | 4 | 5 | 92.2 | 🗸 |
| 36 |  |  |  |  | Renal failure | | 5 | 5 | 4.09 | 3 | 5 | 81.8 | 🗸 |
| 37 |  |  |  |  | Metabolic syndrome | | 4 | 4 | 3.95 | 3 | 5 | 79 | 🗸 |
| 38 |  |  |  |  | Other diseases | | 5 | 5 | 4.38 | 3.5 | 5 | 87.6 | 🗸 |
| 39 |  |  |  |  | Family history | | 5 | 5 | 4.61 | 4 | 5 | 92.2 | 🗸 |
| 40 |  |  |  |  | Smoking | | 5 | 5 | 4.09 | 3 | 5 | 81.8 | 🗸 |
| 41 |  |  |  |  | Hookah smoking | | 4 | 4 | 3.95 | 3 | 5 | 79 | 🗸 |
| 42 |  |  |  |  | Alcohol use | | 3 | 3 | 3.76 | 3 | 5 | 75.2 | 🗸 |
| 43 |  | | Lifestyle | | Medications | | 4 | 4 | 3.86 | 2.5 | 5 | 76.4 | 🗸 |
| 44 |  |  |  |  | Type of medications used | | 5 | 5 | 4.86 | 5 | 5 | 97.2 | 🗸 |
| 45 |  |  |  |  | Route of administration | | 5 | 5 | 4.95 | 5 | 5 | 99 | 🗸 |
| 46 |  |  |  |  | Income level | | 5 | 5 | 4.38 | 3.5 | 5 | 87.6 | 🗸 |
| 47 |  |  |  |  | Body mass index | | 5 | 5 | 4.71 | 4 | 5 | 94.2 | 🗸 |
| 48 |  | | | | Aspirin | | 5 | 5 | 4.86 | 5 | 5 | 97.2 | 🗸 |
| 49 |  |  |  |  | Clopidogrel | | 5 | 5 | 4.95 | 5 | 5 | 99 | 🗸 |
| 50 |  |  |  |  | Warfarin | | 5 | 5 | 4.61 | 4 | 5 | 92.2 | 🗸 |
| 51 | Medications | | | | Beta-blockers | | 5 | 5 | 4.38 | 3.5 | 5 | 87.6 | 🗸 |
| 52 |  |  |  |  | Angiotensin-converting-enzyme inhibitors | | 5 | 5 | 4.86 | 5 | 5 | 97.2 | 🗸 |
| 53 |  |  |  |  | Angiotensin receptor blockers | | 5 | 5 | 4.95 | 5 | 5 | 99 | 🗸 |
| 54 |  |  |  |  | Statin | | 5 | 5 | 4 | 3 | 5 | 80 | 🗸 |
| 55 |  |  |  |  | Non-vitamin K oral anticoagulants (NOACs) | | 5 | 5 | 4.95 | 5 | 5 | 99 | 🗸 |
| 56 |  |  |  |  | Glycoprotein IIb/IIIa inhibitors | | 5 | 5 | 4.86 | 5 | 5 | 97.2 | 🗸 |
| 57 |  |  |  |  | Fibrinolytic inhibitors | | 4 | 4 | 3.95 | 3 | 5 | 79 | 🗸 |
| 58 |  |  |  |  | Narcotics | | 4 | 4 | 3.95 | 3 | 5 | 79 | 🗸 |
| 59 |  |  |  |  | Low-molecular-weight heparin | | 5 | 5 | 4.38 | 3.5 | 5 | 87.6 | 🗸 |
| 60 |  |  |  |  | Unfractionated Heparin | | 5 | 5 | 4.38 | 3.5 | 5 | 87.6 | 🗸 |
| 61 |  |  |  |  | Other medications | | 5 | 4 | 4 | 3 | 5 | 80 | 🗸 |
| 62 | Laboratory test results | | | | Hemoglobin | | 5 | 5 | 4.57 | 4 | 5 | 91.4 | 🗸 |
| 63 |  |  |  |  | Platelets | | 5 | 5 | 4.71 | 5 | 5 | 94.2 | 🗸 |
| 64 |  |  |  |  | Complete blood cells (WBC, RBC, and Hb) | | 5 | 5 | 4.86 | 5 | 5 | 97.2 | 🗸 |
| 65 |  |  |  |  | Prothrombin time with the international normalized ratio (PT-INR) | | 5 | 5 | 4.71 | 5 | 5 | 94.2 | 🗸 |
| 66 |  |  |  |  | Glucose (BS/FBS) | | 5 | 5 | 4.48 | 4 | 5 | 89.6 | 🗸 |
| 67 |  |  |  |  | HBA1C | | 5 | 5 | 4.90 | 5 | 5 | 98 | 🗸 |
| 68 |  |  |  |  | K | | 5 | 5 | 4.57 | 4 | 5 | 91.4 | 🗸 |
| 69 |  |  |  |  | Mg | | 5 | 5 | 4.47 | 4 | 5 | 89.4 | 🗸 |
| 70 |  |  |  |  | Ca | | 5 | 5 | 4.57 | 4 | 5 | 91.4 | 🗸 |
| 71 |  |  |  |  | Na | | 5 | 5 | 4.47 | 4 | 5 | 89.4 | 🗸 |
| 72 |  |  |  |  | Blood urea nitrogen | | 5 | 5 | 4.57 | 4 | 5 | 91.4 | 🗸 |
| 73 |  |  |  |  | Glomerular filtration rate | | 5 | 5 | 4.57 | 4 | 5 | 91.4 | 🗸 |
| 74 |  |  |  |  | C-reactive protein | | 5 | 5 | 4.38 | 4 | 5 | 87.6 | 🗸 |
| 75 |  |  |  |  | Creatine phosphokinase-MB | | 5 | 5 | 4.42 | 4 | 5 | 88.4 | 🗸 |
| 76 |  |  |  |  | Troponin I | | 5 | 5 | 4.38 | 4 | 5 | 87.6 | 🗸 |
| 77 |  |  |  |  | Troponin T | | 5 | 5 | 4.71 | 4 | 5 | 87.6 | 🗸 |
| 78 |  |  |  |  | Cholesterol | | 5 | 5 | 4.71 | 5 | 5 | 94.2 | 🗸 |
| 79 |  |  |  |  | Triglycerides | | 5 | 5 | 4.71 | 5 | 5 | 94.2 | 🗸 |
| 80 |  |  |  |  | Low-density lipoprotein | | 5 | 5 | 4.71 | 5 | 5 | 94.2 | 🗸 |
| 81 |  |  |  |  | High-density lipoprotein | | 5 | 5 | 4.71 | 5 | 5 | 94.2 | 🗸 |
| 82 |  |  |  |  | Erythrocyte sedimentation rate | | 5 | 5 | 4.90 | 5 | 5 | 98 | 🗸 |
| 83 |  |  |  |  | Uric acid | | 5 | 5 | 4.38 | 3.5 | 5 | 87.6 | 🗸 |
| 84 |  |  |  |  | Other tests | | 4 | 4 | 4.33 | 4 | 5 | 86.6 | 🗸 |
| 85 | Physician examination results | | | Present symptoms | Date and time of admission | | 4 | 4 | 4.07 | 4 | 5 | 81.4 | 🗸 |
| 86 |  |  |  |  | Non-specific cerebral palsy | | 5 | 5 | 4.71 | 4 | 5 | 94.2 | 🗸 |
| 87 |  |  |  |  | Dyspnea on exertion | | 5 | 5 | 4.52 | 4 | 5 | 90.4 | 🗸 |
| 88 |  |  |  |  | Typical pain | | 5 | 5 | 4.52 | 4 | 5 | 90.4 | 🗸 |
| 89 |  |  |  |  | Atypical pain | | 5 | 5 | 4.52 | 4 | 5 | 90.4 | 🗸 |
| 90 |  |  |  |  | Dyspnea | | 5 | 5 | 4.07 | 4 | 5 | 81.4 | 🗸 |
| 91 |  |  |  |  | Vital signs (BP, HR, RR, and O_2_ sat) | | 5 | 4 | 4 | 4 | 5 | 80 | 🗸 |
| 92 |  |  |  |  | Electrocardiography results | | 4 | 4 | 4.33 | 4 | 5 | 86.6 | 🗸 |
| 93 |  |  |  |  | Echocardiography results^a^ | | 5 | 5 | 4.47 | 4 | 5 | 89.4 | 🗸 |
| 94 |  |  |  |  | Other signs | | 4 | 4 | 3.76 | 3 | 4 | 76.2 | 🗸 |
| 95 |  |  |  | CAD type detection | | | 5 | 5 | 4.90 | 5 | 5 | 98 | 🗸 |
| 96 | Information on angiography | | | | Date and time of performance | | 3 | 4 | 3.81 | 3 | 5 | 76 | 🗸 |
| 97 |  |  |  |  | Phyisician’s name | | 5 | 5 | 4.80 | 5 | 5 | 96 | 🗸 |
| 98 |  |  |  |  | Number of involved vessels (i.e., SVD, 2VD, and 3VD) | | 5 | 5 | 4.71 | 4 | 5 | 94.2 | 🗸 |
| 99 |  |  |  |  | Angio type, including radial and femoral types | | 5 | 5 | 4.66 | 4 | 5 | 93.2 | 🗸 |
| 100 |  |  |  |  | Involved vessel type (LAD, RCA, LCX, and other branches) | | 5 | 5 | 4.76 | 4.5 | 5 | 95.2 | 🗸 |
| 101 |  |  |  |  | Severity of stenosis (<50%, 50-70%, 70-90%, 90-99%, and 100%) | | 5 | 5 | 4.61 | 4 | 5 | 92.2 | 🗸 |
| 102 |  |  |  |  | Angiography results, indicating mild CAD, moderate CAD, severe vessel occlusion, coronary arterial ectasia, washout, and muscle bridge | | 5 | 5 | 4.66 | 4 | 5 | 93.2 | 🗸 |
| 103 | Non-invasive procedures | | CT angiography | | Coronary artery calcium score | | 5 | 4 | 4.92 | 4 | 5 | 85.7 | 🗸 |
| 104 |  |  |  |  | Dx | | 5 | 4 | 4.29 | 4 | 5 | 85.7 | 🗸 |
| 105 |  |  | Single-photon emission computed tomography-myocardial perfusion imaging | | | | 5 | 4 | 4.29 | 4 | 5 | 85.7 | 🗸 |
| 106 |  |  | Stress or exercise tolerance test, with negative, positive, false negative, and false positive results | | | | 5 | 4 | 4.29 | 4 | 5 | 85.7 | 🗸 |
| 107 |  |  | Echocardiographic results | | | | 5 | 4 | 4.29 | 4 | 5 | 85.7 | 🗸 |
| 108 | Information on invasive  and surgical procedures | | Date of procedure | | | | 3 | 4 | 3.81 | 3 | 5 | 76 | 🗸 |
| 109 |  |  | Time of procedure | | | | 3 | 4 | 3.81 | 3 | 5 | 76 | 🗸 |
| 110 |  |  | Procedure type  (radial/femoral) | | | | 4 | 4 | 4.33 | 4 | 5 | 86.6 | 🗸 |
| 111 |  |  | Type of internvetion (e.g., diagnostic/PCI) | | | | 5 | 5 | 4.57 | 4 | 5 | 91.4 | 🗸 |
| 112 |  |  | Outcomes | | | | 5 | 5 | 4.76 | 4.5 | 5 | 95.2 | 🗸 |
| 113 |  |  | Determination of the type of PCI procedure and related information | | | IIbIIIa injection | 5 | 5 | 4.57 | 4 | 5 | 91.4 | 🗸 |
| 114 |  |  |  |  |  | Thrombectomy | 5 | 5 | 4.42 | 4 | 5 | 88.4 | 🗸 |
| 115 |  |  |  |  |  | Percutaneous old balloon angioplasty | 5 | 5 | 4.57 | 4 | 5 | 91.4 | 🗸 |
| 116 |  |  |  |  |  | Stent insertion (along with documetnation of stent and vessel number) | 5 | 5 | 4.71 | 4 | 5 | 94.2 | 🗸 |
| 117 |  |  |  |  |  | Chronic total occlusion procedure | 5 | 5 | 4.57 | 4 | 5 | 91.4 | 🗸 |
| 118 |  |  |  |  |  | Bifurcation procedure | 5 | 5 | 4.43 | 4 | 5 | 88.6 | 🗸 |
| 119 |  |  |  |  |  | TPM procedure | 4 | 4 | 4.33 | 4 | 5 | 86.6 | 🗸 |
| 120 |  |  |  |  |  | Intra-aortic balloon pump procedure | 5 | 5 | 4.57 | 4 | 5 | 91.4 | 🗸 |
| 121 |  |  | Outcomes | | | Stent thrombosis | 5 | 5 | 4.57 | 4 | 5 | 91.4 | 🗸 |
| 122 |  |  |  |  |  | Thrombolysis in myocardial infarction after PCI | 5 | 5 | 4.52 | 4 | 5 | 90.4 | 🗸 |
| 123 |  |  |  |  |  | The no-reflow phenomenon in PCI | 5 | 5 | 4.38 | 3.5 | 5 | 87.6 | 🗸 |
| 124 |  |  |  |  |  | Occurrence of arrhythmia leading to shock during the procedure | 4 | 4 | 4.09 | 3 | 5 | 81.8 | 🗸 |
| 125 |  |  |  |  |  | Identification of the type of arrhythmia (AV and VE) | 4 | 4 | 3.82 | 2.5 | 5 | 76.4 | 🗸 |
| 126 |  |  |  |  |  | Cardiogenic shock | 5 | 5 | 4 | 3 | 5 | 80 | 🗸 |
| 127 |  |  |  |  |  | Cardiovascular accident/stroke | 5 | 4 | 3.95 | 3 | 5 | 78 | 🗸 |
| 128 |  |  |  |  |  | Cardiac tamponade | 4 | 4 | 3.86 | 2.5 | 5 | 76.4 | 🗸 |
| 129 |  |  |  |  |  | Cervical intraepithelial neoplasia | 5 | 5 | 4.57 | 4 | 5 | 91.4 | 🗸 |
| 130 |  |  |  |  |  | Other vascular complications requiring Rx, RBC, or whole blood transfusion | 5 | 5 | 4.81 | 5 | 5 | 96.2 | 🗸 |
| 131 |  |  |  |  |  | A bleeding event within 72 hours | 5 | 5 | 4.52 | 4 | 5 | 90.4 | 🗸 |
| 132 |  |  |  |  |  | Vascular accidents | 5 | 5 | 4.52 | 4 | 5 | 90.4 | 🗸 |
| 133 |  |  |  |  |  | Other outcomes | 4 | 4 | 3.90 | 3 | 4 | 78 | 🗸 |
| 134 | Patient’s status during discharge | | | | | Date of discharge | 3 | 4 | 3.81 | 3 | 5 | 76 | 🗸 |
| 135 |  |  |  |  |  | Length of hospital stay | 3 | 3 | 3.38 | 3 | 4 | 67.6 | × |
| 136 |  |  |  |  |  | Discharge status | 5 | 5 | 4.28 | 4 | 5 | 85.6 | 🗸 |
| 137 |  |  |  |  |  | Medications prescribed at discharge | 5 | 5 | 4.28 | 4 | 5 | 85.6 | 🗸 |
| 138 |  |  |  |  |  | Other information during discharge | 5 | 5 | 4.28 | 4 | 5 | 85.6 | 🗸 |
| 139 | Patient follow-up | Follow-up results and outcomes after discharge up to six months | | | | Mortality status | 3 | 3 | 3.29 | 3 | 4 | 65.6 | × |
| 140 |  |  |  |  |  | Cause of death | 4 | 4 | 4.33 | 4 | 5 | 86.6 | 🗸 |
| 141 |  |  |  |  |  | Repeated myocardial infarction | 5 | 5 | 4.86 | 5 | 5 | 97.2 | 🗸 |
| 142 |  |  |  |  |  | Need for repeat angiography | 5 | 5 | 4.95 | 5 | 5 | 99 | 🗸 |
| 143 |  |  |  |  |  | Stroke | 5 | 5 | 4.38 | 3.5 | 5 | 87.6 | 🗸 |
| 144 |  |  |  |  |  | Re-hospitalization | 5 | 5 | 4.71 | 4 | 5 | 94.2 | 🗸 |
| 145 |  |  |  |  |  | Adherence to the prescribed medication regimen | 5 | 5 | 4.38 | 3.5 | 5 | 87.6 | 🗸 |
| 146 |  |  |  |  |  | Adverse effects | 5 | 5 | 4.52 | 4 | 5 | 90.4 | 🗸 |
| 147 |  |  |  |  |  | Other information | 5 | 5 | 4.43 | 4 | 5 | 88.6 | 🗸 |
| 148 |  | Follow-up of outcomes due to coronary artery disease | | | | Myocardial infarction | 5 | 5 | 4.81 | 5 | 5 | 96.2 | 🗸 |
| 149 |  |  |  |  |  | Stroke | 5 | 5 | 4.52 | 4 | 5 | 90.4 | 🗸 |
| 150 |  |  |  |  |  | Hospitalization for heart failure | 5 | 5 | 4.43 | 4 | 5 | 88.6 | 🗸 |
| 151 |  |  |  |  |  | Vascular regeneration | 5 | 5 | 4.28 | 4 | 5 | 85.6 | 🗸 |
| 152 |  |  |  |  |  | Bleeding due to treatment | 5 | 5 | 4.52 | 4 | 5 | 90.4 | 🗸 |
| 153 |  |  |  |  |  | Others | 5 | 4 | 4.09 | 3 | 5 | 81.8 | 🗸 |
| 154 |  | Follow-up of patient’s adherence to treatment | | | | Use of medications based on instructions | 5 | 5 | 4.33 | 4 | 5 | 86.6 | 🗸 |
| 155 |  |  |  |  |  | Undergoing medical examinations | 5 | 5 | 4.38 | 4 | 5 | 87.6 | 🗸 |
| 156 |  |  |  |  |  | Adherence to non-pharmacological orders | 5 | 5 | 4.52 | 4 | 5 | 90.4 | 🗸 |
| 157 |  |  |  |  |  | Pharmaceutical consultations | 1 | 2 | 1.90 | 1 | 3 | 38 | × |
| 158 |  |  |  |  |  | Others | 3 | 4 | 3.81 | 3 | 5 | 76 | 🗸 |
| 159 |  | Follow-up of patient’s quality of life | | | | Change in lifestyle | 5 | 4 | 4.09 | 3 | 5 | 81.8 | 🗸 |
| 160 |  |  |  |  |  | Change in diet | 4 | 4 | 4.05 | 3 | 5 | 81 | 🗸 |
| 161 |  |  |  |  |  | Others | 3 | 4 | 3.81 | 3 | 5 | 76 | 🗸 |
| 162 |  | Follow-up of death status | | | | Living status | 4 | 4 | 3.52 | 3 | 4 | 70.4 | × |
| 163 |  |  |  |  |  | Cause of death | 4 | 4 | 4.07 | 4 | 5 | 81.4 | 🗸 |
| 164 |  |  |  |  |  | Date and time of death | 3 | 3 | 3.14 | 3 | 4 | 62.8 | × |
| 165 |  |  |  |  |  | Place of death | 3 | 4 | 3.81 | 3 | 5 | 76 | 🗸 |
| 166 |  |  |  |  |  | Other data | 4 | 4 | 3.90 | 3 | 5 | 78 | 🗸 |
